# Expression of miR-652-3p and Effect on Apoptosis and Drug Sensitivity in Pediatric Acute Lymphoblastic Leukemia

**DOI:** 10.1155/2018/5724686

**Published:** 2018-06-05

**Authors:** Qian Jiang, Xiaojing Lu, Pengli Huang, Chao Gao, Xiaoxi Zhao, Tianyu Xing, Gang Li, Shilai Bao, Huyong Zheng

**Affiliations:** ^1^Beijing Key Laboratory of Pediatric Hematology Oncology, National Key Discipline of Pediatrics (Capital Medical University), Key Laboratory of Major Diseases in Children, Ministry of Education, Hematology Oncology Center, Beijing Children's Hospital, Capital Medical University, National Center for Children's Health, Beijing 100045, China; ^2^Maternity and Child Care Hospital of Henan Province, Zhengzhou, Henan 450052, China; ^3^Institute of Genetics and Developmental Biology, Chinese Academy of Sciences, Beijing 100101, China

## Abstract

MicroRNAs (miRNAs) expression profiles were screened in plasma samples from pediatric patients with acute lymphoblastic leukemia (ALL) and healthy controls, using qRT-PCR-based TaqMan low-density miRNA arrays. MiR-652-3p (a circulating miRNA) was downregulated in new diagnosis (ND) patients compared with healthy controls. The levels of miR652-3p were restored in complete remission (CR) but were downregulated again in disease relapse (RE). The expression pattern of miR-652-3p was validated in bone marrow (BM) samples from other pediatric ALL patients. MiR-652-3p was significantly upregulated in BM when the patients (*n*=86) achieved CR, as compared with the matched ND samples (p<0.001). Moreover, the miR-652-3p levels in BM decreased again in two patients at RE. In addition, the lymphoblastic leukemia cell lines Reh and RS4:11 were found to have lower levels of miR-625-3p than the normal B-cell line. Overexpression of miR-652-3p using agomir increased the sensitivity to vincristine and cytarabine (all p<0.05) and promoted apoptosis (both p<0.05) in Reh and RS4:11 cells. In conclusion, the results suggested that a low level of miR-652-3p might be involved in the pathogenesis of pediatric ALL. Overexpression of miR-652-3p might suppress lymphoblastic leukemia cells, promoting apoptosis and increasing sensitivity to chemotherapeutic drugs.

## 1. Introduction

Acute lymphoblastic leukemia (ALL) is the most common pediatric cancer and causes significant morbidity in children [1]. Remarkable progress has been made in the last six decades due to the optimization of risk-directed therapeutic strategies and intensive protocols against ALL [2]. The overall survival rates of patients with ALL now exceed 80%, but still 15-20% of patients will experience relapse [3–6] and the outcomes of the relapsing patients are dismal [7, 8], mainly because the pathogenesis of ALL and its relapse is not fully understood yet. Therefore, exploring the mechanisms of ALL and finding efficient leukemic markers to distinguish the patients who can potentially relapse are warranted.

MicroRNAs (miRNAs) are small noncoding single-strand RNAs of approximately 22 nucleotides [9]. MiRNAs can be either tumor activators or suppressors by binding to the 3' untranslated region of their target mRNA [10]. MiRNAs can lead to mRNA degradation or repress translational [11]. Therefore, they are associated with the regulation of a variety of biological processes such as cell cycle, apoptosis, drug sensitivity, protein transport, and angiogenesis. Many cancers are associated with alterations in the expression levels of miRNAs [12, 13]. A large number of studies have focused on the role of miRNAs in carcinogenesis and ALL development [14] and they demonstrated the role of miRNAs as biomarkers for diagnosis, prognosis, and response to chemotherapy in pediatric ALL. Bone marrow (BM) samples from children diagnosed with ALL revealed that miRNA expression profiles are relevant to the severity of the disease [15, 16]. In addition, an increasing number of studies focus on miRNAs circulating in plasma as noninvasive potential biomarkers that clinically characterize the tumor [17]. Luna-Aguirre et al. performed and validated an expression profile of plasma miRNAs; they found that 77 circulating miRNAs were differentially expressed in ALL and miR-511 was considered the most valuable biomarker for distinguishing B-ALL from normal controls [18].

In this study, we hypothesized that miRNAs take part in the pathophysiology of pediatric ALL. A previous study by our group showed that a number of miRNAs (including miR-223 and miR-27a) were associated with drug resistance and relapse in pediatric ALL [19]. Therefore, the differential expression of miRNAs was first examined in plasma samples from pediatric patients with ALL and normal controls. This analysis showed that miR-652-3p was one of the differentially expressed circulating miRNAs between the pediatric ALL samples and controls, which to our knowledge has not been studied in ALL before. The expression of miR-652-3p was reported in myeloid leukemia, hepatocellular carcinoma, gastric cancer, and nonsmall cell lung cancer [20–23], but its expression pattern is different in different diseases. For example, the expression level reduced in liver disease and hepatocellular carcinoma, indicating a decreased risk of the disease; nevertheless, its expression increased in gastric cancer and nonsmall cell lung cancer, promoting metastasis [20–23].

Next, the expression pattern of miR-652-3p in BM samples of pediatric ALL was examined at new diagnosis (ND), complete remission (CR), and relapse (RE). Then, the biological functions of miR-652-3p in the lymphoblastic leukemia cell lines* TEL/AML1*^+^ Reh and* MLL/AF4*^+^ RS4:11 (both are pre-B-cell lines) were explored. The normal B (NB) cell line derived from Epstein-Barr virus-transformed human B cells was used as the control. The results might provide detailed information on the pathogenesis of ALL and the use of miR-652-3p as a diagnostic marker or even potential therapeutic target for pediatric ALL.

## 2. Materials and Methods

### 2.1. Patients and Clinical Samples

The study was approved by the Clinical Research Ethics Committee of Beijing Children's Hospital. Written informed consent was obtained from the parents of each child involved in this research.

Between February 2010 and July 2013, we collected plasma samples from three pediatric ALL patients (Table 1) at their ND as well as CR and from three other pediatric ALL patients (Table 1) at their RE. In addition, matched BM samples were collected from 86 other pediatric patients with ALL (Table 2) at ND and CR; for three out of those 86 patients (Table 3), BM samples were also obtained at RE. All patients were treated at the Hematology Centre of Beijing Children's Hospital, Capital Medical University. The patients were diagnosed with ALL using a combination of morphology, immunology, cytogenetics, and molecular biology, according to the “Recommendations for diagnosis and treatment of acute lymphoblastic leukemia in childhood (3rd revised version)” [28]. The patients were classified as standard-risk, intermediate-risk, and high-risk groups according to age, WBC count, immunophenotype, cytogenetic and molecular aberrations, prednisone response, morphological remission at the end of induction therapy (based on BFM risk criteria), and minimal residual disease (MRD) at the end of induction therapy and the beginning of consolidation therapy [24–27]. The patients were treated according to the Chinese Children's Leukemia Group 2008 protocol. Plasma samples were collected from three healthy children considered as normal controls (Table 1).

Blood samples were centrifuged at 1500 ×*g* for 10 min at 4°C to collect the supernatant (serum) in RNase-free tubes. TRIzol Reagent (Invitrogen, Carlsbad, CA, USA) was used to isolate mononuclear cells from the BM samples.

### 2.2. MicroRNA Array

The differential expression of the miRNAs was compared among plasma samples from pediatric patients with ALL (ND, CR, and RE) and normal controls. The total RNA of the serum was isolated using a mirVana miRNA isolation kit (Ambion, TX, USA), according to the manufacturer's instructions. The expression profiles were examined with highly standardized quantitative real-time polymerase chain reaction (qRT-PCR) based TaqMan low-density microRNA arrays (Applied Biosystems, CA, USA). Megaplex RT Primers, Human Pool Set v3.0 (Cat. 4444745), and TaqMan microRNA reverse transcription kit (Cat. 4366596) were used for cDNA preparation. TaqMan Array Human MicroRNA A+B Cards Set v3.0 (Cat. 4444913) was used to quantify 754 human miRNAs and three endogenous controls for data normalization. The manipulation was carried out in accordance with the instructions.

### 2.3. Quantitative Real-Time PCR

The expression of miR-652-3p was measured with cDNA isolated from BM samples or cell lines. Endogenous U6 was used as an internal control. Total RNA was extracted using TRIzol, according to the manufacturer's instructions. The cDNA was prepared with the TaqMan microRNA reverse transcription kit using specific primers for miR-652-3p or U6 picked from the Megaplex RT Primers, Human Pool Set v3.0. The 20-*μ*L PCR reaction mixture included 8 *μ*L of nuclease-free water, 1 *μ*L of cDNA, 10 *μ*L of TaqMan Universal PCR Master Mix, and 1 *μ*L of specific primers. Real-time PCR was performed using the Applied Biosystems 7500 Sequence Detection System. The initial denaturation was performed at 95°C for 10 min, followed by 45 cycles at 95°C for 15 s and 95°C for 1 min. The Ct values (Ct) were calculated with the SDS 2.0.5 software (Applied Biosystems, CA, USA) using the automatic threshold setting. The experiments were run in triplicate, and average Ct was calculated. The average expression level of miR-652-3p was normalized to U6 using the 2^-△△CT^ method.

### 2.4. Cell Culture

The* TEL/AML1*^+^ Reh and* MLL/AF4*^+^ RS4:11 cell lines (Cell Bank of the Chinese Academy of Science, Shanghai, China) and NB cell line (BaoShilai, Institute of Genetics and Developmental Biology, Chinese Academy of Sciences) were cultured in RPIM-1640 medium (Gibco, NY, USA) supplemented with 10% fetal bovine serum (FBS) (Gibco, NY, USA) in a 5% CO_2_ humidified atmosphere at 37°C.

### 2.5. Cell Transfection

The miR-652-3p agomir (agomir-652) and its negative control mismatched miR-652-3p agomir (agomir-Ctrl) were synthesized by Ribobio (Guangdong, China). The miR-652-3p agomir (50nM) and agomir-Ctrl (50nM) were transfected into the Reh and RS4:11 cell lines using the X-treme GENE HP DNA Transfection Reagent (Roche Diagnostics, Penzberg, Germany), according to the manufacturer's instructions. After transfection, the cells were incubated in 2 mL of antibiotic-free media containing 10% FBS for 48h. Then, drug sensitivity and cell apoptosis assays were performed.

### 2.6. Drug Sensitivity Assay

Vincristine (VCR; Cat. MB20820, Meilunbio Technologies Co., Ltd., Dalian, China) and cytarabine (Ara-C; Cat. MB20226; Meilunbio Technologies Co., Ltd.) were added to the cells. The negative control was cells without treatment. The drug sensitivity was determined using the CellTiter 96 AQueous One Solution Cell Proliferation assay (MTS) (Promega, WI, USA), according to the manufacturer's instructions, after having treated the cells with the drugs for 24h. The relative cell viability under drug treatment was calculated at each drug concentration: (OD drug at certain concentration)/(OD of negative control) × 100%. Then, the growth inhibition curve was drawn. The 50% inhibitory concentration (IC50) was used as the measure of cellular resistance to each drug. The IC50 was calculated using the GraphPad Prism 6.0 software (www.graphpad.com).

### 2.7. Cell Apoptosis Assay

The cells were collected and washed twice with cold phosphate-buffered saline. The apoptotic cells were detected using an Annexin V-fluorescein isothiocyanate/propidium iodide double staining apoptosis detection kit (BD Biosciences, CA, USA) and the percentage of apoptotic cells was analyzed using flow cytometry (Becton Dickinson FACS Canto II), according to the manufacturer's instructions (BD Biosciences).

### 2.8. Statistical Analysis

Data were analyzed using SPSS 16.0 (IBM, Armonk, NY, USA). Categorical data were expressed as a percentage (%). Continuous data with normal distribution were expressed as mean ± standard deviation and analyzed using Student's* t*-test between two groups or using analysis of variance among multiple groups. Continuous data with nonnormal distribution were expressed as median (range) and analyzed using the nonparametric Wilcoxon test. Two-sided* P* values <0.05 were considered statistically significant.

## 3. Results

### 3.1. Expression of miR-652-3p Was Negatively Correlated with the Progression of Pediatric ALL

**Figure 1:**
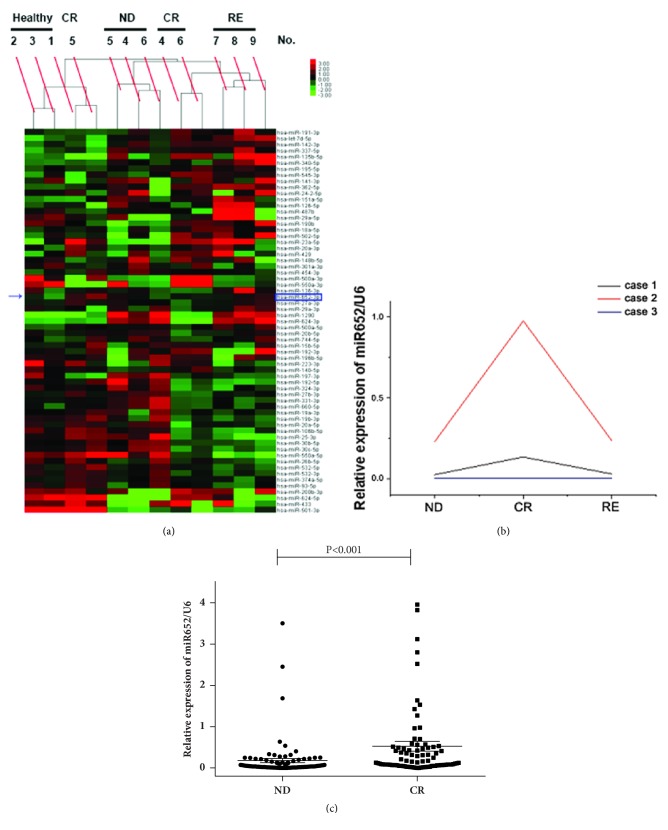
Expression pattern of miR-652-3p in plasma and bone marrow (BM) samples. (a) Hierarchical clustering of three healthy controls (No. 1-3), three pediatric patients with acute lymphoblastic leukemia (ALL) (No. 4-6) at their new diagnosis (ND) or complete remission (CR), and another three pediatric patients with ALL (No. 7-9) at relapse (RE), based on the expression profile of circulating miRNAs, as examined by qRT-PCR-based TaqMan low-density miRNA arrays. The expression pattern of miR-652-3p is indicated by a blue arrow. (b) BM samples were obtained from three pediatric patients with ALL apart from the ones in (a) at ND, CR, and RE. The changes in the expression of miR-652-3p were detected by qPCR. (c) Sample size in (b) was enlarged to 86 patients, and the expression of miR-652-3p in BM was analyzed at ND and CR.

The expression profiles of 754 miRNAs in plasma samples from pediatric patients with ALL and healthy controls were analyzed using qRT-PCR-based TaqMan low-density miRNA arrays, in order to identify miRNAs differentially expressed in pediatric ALL. We uploaded the miRNA array results as the GEO number GSE109868. Hierarchical clustering was performed based on the expression profiles of circulating miRNAs for the three healthy controls, three pediatric patients with ALL at ND or CR, and three other pediatric patients with ALL at RE (Figure 1(a)). Setting* P*<0.05 and false discovery rate <0.05 as the criteria, 45 miRNAs were found to be differentially expressed between the healthy controls and patients at ND; 21 miRNAs were differentially expressed both from ND to CR and from CR to RE (Supplementary Table 1). Among the differentially expressed miRNAs, miR-652-3p was significantly lower in patients at ND compared with healthy controls; miR-652-3p levels were restored at CR but downregulated again at RE (all* P*<0.05, Supplementary Table 1). Therefore, miR-652-3p was selected for further study because it was not previously studied in ALL.

Next, the expression of miR-652-3p was validated in BM samples from another three pediatric patients with ALL using qRT-PCR. For the paired BM samples collected from the same patients at ND, CR, and RE, the expression of miR-652-3p in two patients showed an obvious increase from ND to CR, but miR-652-3p decreased again at RE (Figure 1(b)). Subsequently, the sample size was enlarged to a total of 86 pediatric patients with ALL in order to compare the expression of miR-652-3p in BM at ND and CR. The results showed that the expression level of miR-652-3p was significantly upregulated in BM samples when the patients achieved CR compared with the matched ND samples (*P*<0.001, Figure 1(c)).

### 3.2. Overexpression of miR-652-3p Increased the Sensitivity of Lymphoblastic Leukemia Cells to Chemotherapeutic Drugs

**Figure 2:**
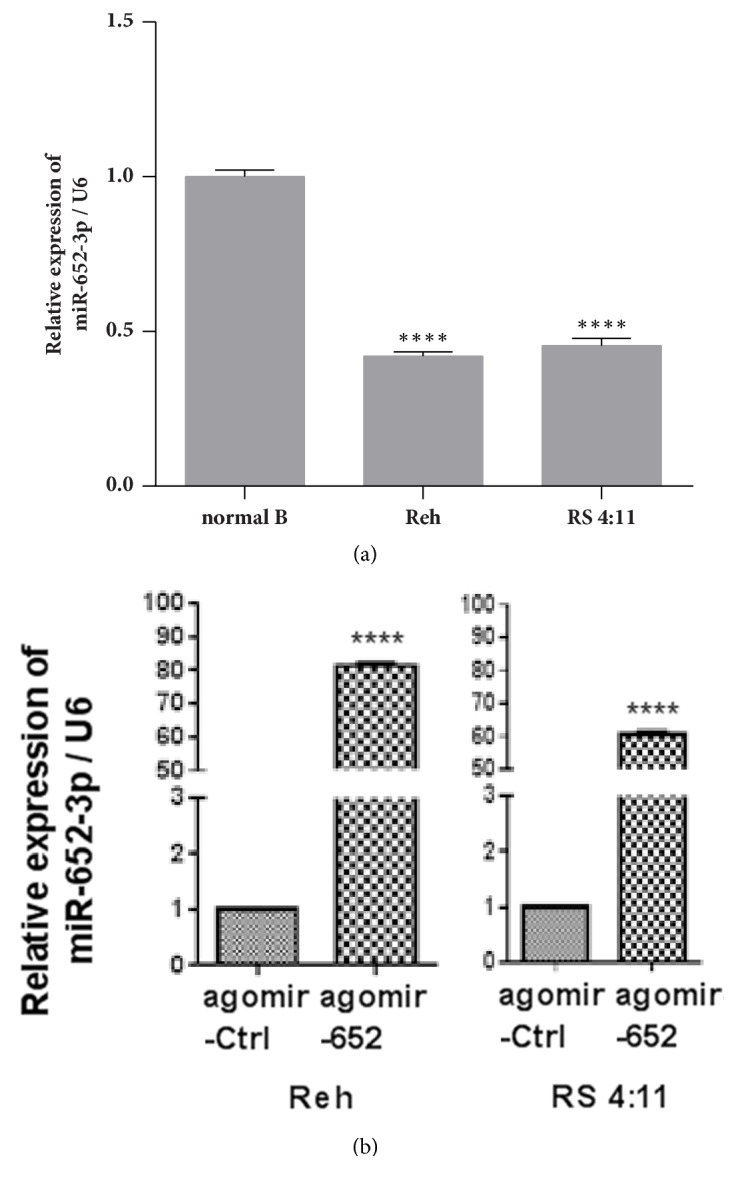
Overexpression of miR-652-3p in lymphoblastic leukemia cell lines. (a) Endogenous expression levels of miR-652-3p were detected in normal B cells and the lymphoblastic leukemia cell lines Reh and RS4:11 using qPCR. *∗∗∗∗P*<0.0001 vs. normal B cells. (b) Overexpression of miR-652-3p using agomir in Reh cells (left) and RS4:11 cells (right) was confirmed by qPCR. Agomir-Ctrl: cells transfected with control agomir; agomir-652: cells transfected with miR-652-3p agomir. *∗∗∗∗P*<0.0001 vs. agomir-Ctrl.

The clinical results suggested that relatively lower expression of miR-652-3p was associated with poorer outcome of pediatric ALL, implying the possibility that miR-652-3p could affect the response to chemotherapy in ALL. To verify this possibility, the expression of miR-652-3p in the Reh, RS4:11, and NB cell lines was assessed. A significantly lower expression of miR-652-3p was observed in the Reh and RS4:11 cell lines compared with the NB cell line (*P*<0.0001, Figure 2(a)). Then, the miR-652-3p agomir was successfully overexpressed in the Reh and RS4:11 cell lines to explore whether the modulation of miR-652-3p expression changed the features of lymphoblastic leukemia cells (Figure 2(b)).

**Figure 3:**
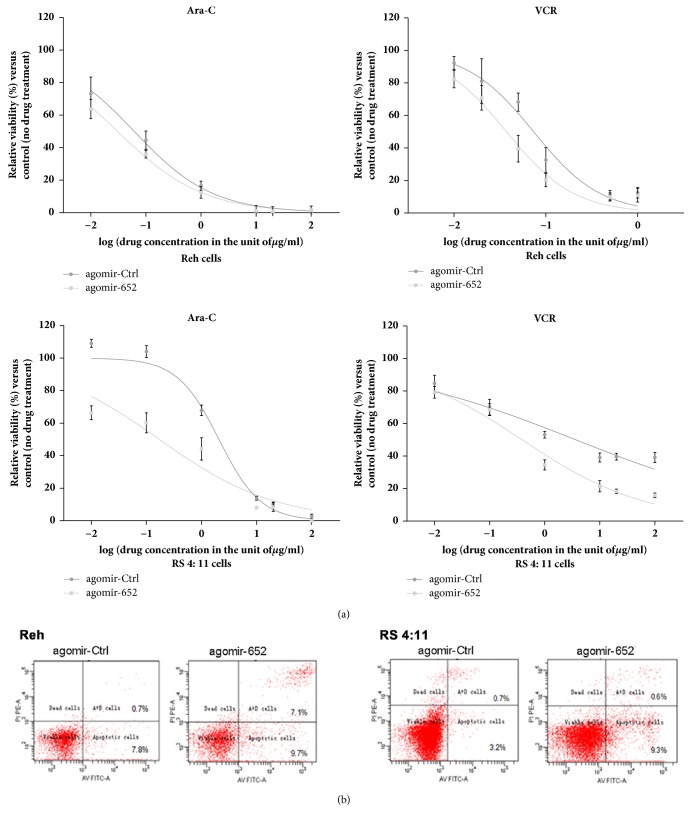
Effects of overexpressing miR-652-3p in lymphoblastic leukemia cell lines. (a) The growth of cells was examined using the MTS assay after drug treatment for 24h. For the Reh cell line treated with cytarabine (Ara-C), the IC50 of agomir-Ctrl was 0.06129 *μ*g/mL and the IC50 of agomir-652 was 0.03049 *μ*g/mL. For the Reh cell line treated with vincristine (VCR), the IC50 of agomir-Ctrl was 0.07354 *μ*g/mL and the IC50 of agomir-652 was 0.03736 *μ*g/mL. For the RS 4:11 cell line treated with Ara-C, the IC50 of agomir-Ctrl was 2.074 *μ*g/mL and the IC50 of agomir-652 was 0.1715 *μ*g/mL (panel 3). For the RS 4:11 cell line treated with VCR, the IC50 of agomir-Ctrl was 3.665 *μ*g/mL and the IC50 of agomir-652 was 0.3891 *μ*g/mL. (b) Representative images of FACS analysis of apoptosis in the Reh (left) and RS4:11 (right) cell lines.

The sensitivity to VCR and Ara-C was significantly higher in cells transfected with miR-652-3p agomir compared with cells transfected with control agomir, as indicated by reduced IC50 (all* P*<0.05, Figure 3(a)). These results suggested that increasing the expression level of miR-652-3p could enhance the sensitivity of lymphoblastic leukemia cells to chemotherapeutic drugs.

### 3.3. Overexpression of miR-652-3p Promoted the Apoptosis of Lymphoblastic Leukemia Cells

Besides, the effect of miR-652-3p on cell apoptosis was analyzed using flow cytometry. The results showed that miR-652-3p agomir significantly promoted the apoptosis of the Reh and RS4:11 cells (Figure 3(b)), suggesting that miR-652-3p might increase the apoptosis of lymphoblastic leukemia cells, resulting in growth inhibition.

## 4. Discussion

This study was performed to investigate the expression profile of miRNAs in pediatric patients with ALL to validate their use as biomarkers and potential therapeutic targets. Cimmino et al. first reported that miR-15 and miR-16 (located in a cluster at 13q14.3) were downregulated in about 65% of patients with B-cell chronic lymphoblastic leukemia. Since then, many studies focused on the relationship between the hematological malignancies and miRNAs [29], but few studies have been performed on the role of miRNAs in childhood ALL. This line of research can help understand the pathogenesis and biological process of childhood ALL. MiR-708 was more highly expressed in* TEL-AML1, BCR-ABL, E2A-PBX1*, hyperdiploid, and B-other cases than in* MLL*-rearranged and T-ALL cases. The expression level of miR-196b was higher in* MLL*-rearranged cases than in the remaining precursor B-ALL cases [15]. These findings pointed out that different miRNAs might be specifically involved in different subtypes of pediatric ALL. The expression profile of miRNAs showed significant associations between the expression level of miR-196b and T-ALL and miR-100 and low white blood cell counts at ND [16]. These studies highlighted the role of miRNAs in childhood ALL by examining BM samples. Besides, some studies focused on the circulating miRNAs in cancer [17]. Luna-Aguirre et al. detected the expression profile of circulating miRNAs in childhood B-cell ALL. The results revealed that 77 circulating miRNAs were differentially expressed: miR-511, miR-222, and miR-34a were overexpressed, while miR-199a-3p, miR-223, miR-221, and miR-26a were downregulated, compared with the healthy controls [18].

The present study evaluated the expression of miR-652-3p in plasma and BM samples from pediatric patients with ALL for the first time. MiR-652-3p has recently been identified as a tumor-related gene, but how miR-652-3p is involved in cancer initiation and progression is still largely unknown. MiR-652-3p is upregulated in osteosarcoma, rectal cancer, nonsmall cell lung cancer, and breast cancer but downregulated in malignant pleural mesothelioma [23, 30–33]. In the present study, microRNA array analysis showed a decrease in the miR-652-3p levels in the plasma of pediatric patients with ALL. The expression pattern was validated in BM samples, confirming that the expression levels were lower at ND than at CR. Furthermore, the BM levels of miR-652-3p decreased again at RE in two of three patients. A previous study on pediatric ALL demonstrated that the expression levels of miR-511 was associated with the prognosis of pediatric ALL [33]. The number of patients with available follow-up information in the present study was too small to provide information on the prognostic value of miR-652-3p in ALL. Hence, this needs to be validated using larger samples.

One of the most important reasons for poor treatment response of pediatric ALL is drug resistance. A series of studies suggested that miRNAs are associated with drug resistance [34–37]. VCR and Ara-C are commonly used chemotherapeutic drugs for treating ALL and they are cell cycle-specific drugs [38, 39]. VCR acts by destabilizing microtubules, while Ara-C acts by damaging DNA during the S phase of the cell cycle [40, 41]. Ara-C has been shown to increase apoptosis in G0 B-chronic lymphocytic leukemia cells [41–43]. The present study suggests that the expression of miR-652-3p is markedly lower in the lymphoblastic leukemia cell lines Reh and RS4:11 compared with the NB cell line. The overexpression of miR-652-3p in the Reh and RS4:11 cell lines using agomir indicated that the resistance to VCR and Ara-C increased to different degrees depending on the drug and the cell line, with a greater sensitivity induced in RS4:11 cells upon Ara-C treatment. In addition, apoptosis in these two cell lines was promoted by the overexpression of miR652-3p. These findings suggest that increased levels of miR-652-3p might assist in suppressing lymphoblastic leukemia cells. They also implied that the increase in apoptosis induced by the overexpression of miR652-3p might, to some extent, contribute to the increased sensitivity of lymphoblastic leukemia cells to chemotherapeutic drugs. Unfortunately, the target genes of miR-652-3p, which might be involved in drug resistance, are not identified for now, but the possible biological targets were predicted (Supplementary Table 2) using the TargetScan software (Supplementary Table 3), the microRNA.org database (Supplementary Table 4), and miRDB database (Supplementary Table 5).

Overall, the study is preliminary at this stage and the results need to be validated, especially with additional numbers of RE patients. More experiments, such as the TUNEL assay, need to be performed to confirm the effects of miR-652-3p on lymphoblastic leukemia cells. Nevertheless, the results suggested that unlike the case in some cancers, increased miR-652-3p in ALL is likely to be beneficial to patients.

## 5. Conclusions

In summary, miR-652-3p appeared to be a potentially useful biomarker for pediatric ALL because of the specific expression of miR-652-3p at ND, CR, and RE. Furthermore, the present study provided evidence that miR-652-3p might play as cancer-suppressive functions in pediatric ALL, regulating apoptosis, and resistance to chemotherapeutic drugs.

## Figures and Tables

**Table 1:** Baseline characteristics of healthy children and pediatric patients with ALL (serum samples).

No	Sample type	Sex	Age (at ND)	Chromosome	CR duration
1	Healthy control	Male	3	None	- -
2	Healthy control	Female	9	None	- -
3	Healthy control	Female	12	None	- -
4	ND and matched CR	Male	4	t(12;21)	- -
5	ND and matched CR	Female	11	None	- -
6	ND and matched CR	Female	8	None	- -
7	RE	Male	3	t(12;21)	23 months
8	RE	Female	7	None	31 months
9	RE	Female	12	None	43 months

ALL: acute lymphoblastic leukemia; CR: complete remission; ND: new diagnosis; RE: relapse

**Table 2:** Clinical characteristics of pediatric patients with ALL (BM samples).

**Sex**	*Male*	64.0% (*n* = 55)
	*Female*	36.0% (*n* = 31)
**Age (at ND)**		5.00 ± 2.89
**WBC number (at ND)**		12.41 (1.74–316)
**Blast cells in CSF **	*Negative*	94.2% (*n* = 81)
**(at ND)**	*CNS2*	2.4% (*n* = 2)
	*CNS3*	3.5% (*n* = 3)
**Percentage of blast cells in BM puncture (at ND)**		91.46 ± 6.72%
**Immunophenotyping**	*Common B cell*	86.0% (*n* = 74)
	*Pre B cell*	7.0% (*n* = 6)
	*Pre B cell*	5.8% (*n* = 6)
**Fusion gene**	*BCR-ABL*	2.3% (*n* = 2)
	*E2A-PBX1*	14.0% (*n* = 12)
	*TEL-AML1*	20.3% (*n* = 23)
**Risk stratification**	*Standard-risk*	33.7% (*n* = 29)
	*Intermediate-risk*	55.8% (*n* = 48)
	*High-risk*	10.5% (*n* = 9)
**CNS involvement**	*Yes*	8.1% (*n* = 7)
	*No*	91.9% (*n* = 79)
**Prednisone response on Day 8**	*Favorable*	97.7% (*n* = 84)
	*Poor* ^1^	2.0% (*n* = 2)
**BM response on day 15 by morphology**	*Non-remission*	7.0% (*n* = 6)
	*PR*	11.6% (*n* = 10)
	*CR*	81.4% (*n* = 70)
**BM response on day 33 by morphology**	*Non-remission*	1.2% (n=1)
	*PR* ^2^	1.2% (n=1)
	*CR*	97.7% (n=84)

ALL: acute lymphoblastic leukemia; BM: bone marrow; CR: complete remission; CNS: central nervous system; CSF: cerebrospinal fluid; WBC: white blood cell; ND: new diagnosis; PR: partial remission.

The patients were classified as standard-risk, intermediate-risk, and high-risk groups according to age, WBC count, immunophenotype, cytogenetic and molecular aberrations, prednisone response, morphological remission at the end of induction therapy (based on BFM risk criteria), and minimal residual disease (MRD) at the end of induction therapy and the beginning of consolidation therapy [24–27].

^1^When blasts are <1000/*µ*l.

^2^The patient with PR on day 33 finally achieved CR 6 months after admission to the hospital.

**Table 3:** Clinical characteristics of the three pediatric patients with ALL^1^ who relapsed (BM samples).

	Case 1	Case 2	Case 3
**Sex**	Male	Male	Male
**Age (at ND)**	4.3	5.11	8.4
**WBC number (at ND)**	69.67	127	27.75
**Blast cells in CSF (at ND)**	Negative	Negative	Negative
**Percentage of blast cells in BM puncture (at ND)**	97%	92%	95%
**Immunophenotyping**	Common B cell	Common B cell	Common B cell
**Fusion gene**	*TEL-AML1*	*TEL-AML1*	None
**Risk stratification**	Intermediate-risk	Intermediate-risk	Intermediate-risk
**CNS involvement**	Yes	No	No
**Prednisone response on Day 8**	Favorable	Favorable	Favorable
**BM response on Day 15 by morphology**	CR	PR	CR
**BM response on Day 33 by morphology**	CR	CR	CR
**CR duration (months)**	41	53	26

ALL: acute lymphoblastic leukemia; BM: bone marrow; CR: complete remission; CNS: central nervous system; CSF, cerebrospinal fluid; WBC: white blood cell; ND: new diagnosis; PR: partial remission.

The patients were classified as standard-risk, intermediate-risk, and high-risk groups according to age, WBC count, immunophenotype, cytogenetic and molecular aberrations, prednisone response, morphological remission at the end of induction therapy (based on BFM risk criteria), and minimal residual disease (MRD) at the end of induction therapy and the beginning of consolidation therapy [24–27].

^1^These three patients were from the 86 patients whose details are summarized in Table 2.

## Supplementary Materials

Supplementary Materials Supplementary Table 1: the expression profiles of circulating miRNAs were analyzed and compared among healthy controls and pediatric patients with ALL at new diagnosis (ND), complete remission (CR), and relapse (RE), using the qRT-PCR-based TaqMan low-density miRNA arrays. Supplementary Table 2: summary of the target prediction for miR-652-3p. Supplementary Table 3: the possible biological targets of miR-652-3p predicted by Target Scan software. Supplementary Table 4: the possible biological targets of miR-652-3p predicted by microRNA.org database. Supplementary Table 5: the possible biological targets of miR-652-3p predicted by miRDB database.
