# Supplementary material for: Expression of miR-652-3p and Effect on Apoptosis and Drug Sensitivity in Pediatric Acute Lymphoblastic Leukemia
Source: Biomed Res Int. 2018 Jun 5;2018:5724686. doi: 10.1155/2018/5724686 (PMC6008837; doi:10.1155/2018/5724686)
Supplement: Supplementary Materials — Supplementary Table 1: the expression profiles of circulating miRNAs were analyzed and compared among healthy controls and pediatric patients with ALL at new diagnosis (ND), complete remission (CR), and relapse (RE), using the qRT-PCR-based TaqMan low-density miRNA arrays. Supplementary Table 2: summary of the target prediction for miR-652-3p. Supplementary Table 3: the possible biological targets of miR-652-3p predicted by Target Scan software. Supplementary Table 4: the possible biological targets of miR-652-3p predicted by microRNA.org database. Supplementary Table 5: the possible biological targets of miR-652-3p predicted by miRDB database. [file 5724686.f1.zip › Concise description of supplementary materials.docx]

Supplementary Table 1: The expression profiles of circulating miRNAs were analyzed and compared among healthy controls and pediatric patients with ALL at new diagnosis (ND), complete remission (CR) and relapse (RE), using the qRT-PCR-based TaqMan low-density miRNA arrays.

Supplementary Table 2: Summary of the target prediction for miR-652-3p.

Supplementary Table 3: The possible biological targets of miR-652-3p predicted by Target Scan software.

Supplementary Table 4: The possible biological targets of miR-652-3p predicted by microRNA.org database.

Supplementary Table 5: The possible biological targets of miR-652-3p predicted by miRDB database.
